# α-Lipoic Acid Maintains Brain Glucose Metabolism *via* BDNF/TrkB/HIF-1α Signaling Pathway in P301S Mice

**DOI:** 10.3389/fnagi.2020.00262

**Published:** 2020-08-21

**Authors:** Yan-hui Zhang, Xin-zhu Yan, Shuang-feng Xu, Zhong-qiu Pang, Lin-bo Li, Yang Yang, Yong-gang Fan, Zhuo Wang, Xin Yu, Chuang Guo, Qiang Ao

**Affiliations:** ^1^School of Fundamental Sciences, China Medical University, Shenyang, China; ^2^College of Life and Health Sciences, Northeastern University, Shenyang, China; ^3^Institute of Health Science, China Medical University, Shenyang, China

**Keywords:** Alzheimer’s disease, α-lipoic acid, hypoxia-inducible factor-1α, glucose metabolism, tau phosphorylation

## Abstract

The microtubule-associated protein tau is closely correlated with hypometabolism in Alzheimer’s disease (AD). α-lipoic acid (LA), which is a naturally occurring cofactor in mitochondrial, has been shown to have properties that can inhibit the tau pathology and neuronal damage in our previous research. However, if LA affects glucose metabolism when it reverses tau pathology remains unclear, especially concerning the potential mechanism. Therefore, we make a further study using the P301S mouse model (a tauopathy and AD mouse model which overexpressing fibrillary tau) to gain a clear idea of the aforementioned problems. Here, we found chronic LA administration significantly increased glucose availability by elevating glucose transporter 3 (GLUT3), GLUT4, vascular endothelial growth factor (VEGF) protein and mRNA level, and heme oxygenase-1 (HO-1) protein level in P301S mouse brains. Meanwhile, we found that LA also promoted glycolysis by directly upregulating hexokinase (HK) activity, indirectly by increasing proliferator-activated receptor gamma coactivator 1-alpha (PGC-1α) and DNA repair enzymes (OGG1/2 and MTH1). Further, we found the underlying mechanism of restored glucose metabolism might involve in the activation of brain-derived neurotrophic factor (BDNF)/tyrosine Kinase receptor B (TrkB)/hypoxia-inducible factor-1α (HIF-1α) signaling pathway by LA treatment.

## Introduction

Alzheimer’s disease (AD) is a growing health burden worldwide. Extracellular amyloid plaques, intracellular neurofibrillary tangles (NFTs), and decreased glucose metabolism are the main characteristics of AD brains (Scheltens et al., 2016; Butterfield and Halliwell, 2019). Recent studies have reported a spatial association between tau and glucose metabolism, the brain region where has the decreased glucose metabolism level is strongly associated with increased tau level in AD patients (Adams et al., 2019; Baghel et al., 2019). Glucose transporters (GLUTs) deficiency and glucose deprivation have been shown to trigger tau hyperphosphorylation and cognitive impairment (Liu et al., 2008; Lauretti and Pratico, 2015; Lauretti et al., 2017), which suggests a strong relationship between hyperphosphorylated tau levels and glucose metabolism in AD progression.

The brain mostly uses glucose for energy, but glucose metabolism is dramatically decreased in AD. Glucose uptake and glycolysis are major factors of glucose metabolism in neurons. However, not only the glucose uptake but also glycolysis is dysregulated in AD (Winkler et al., 2015). Glucose uptake occurs mainly by facilitated diffusion through specific GLUTs (Jais et al., 2016). To date, 14 GLUTs have been detected in mammals, and GLUT1, GLUT3, and GLUT4 play major roles in facilitating brain glucose uptake (McEwen and Reagan, 2004; Simpson et al., 2008). GLUT1 is primarily found in microvascular endothelial and glial cells (Simpson et al., 1999), GLUT3 and GLUT4 are primarily present in neurons (Maher et al., 1994). Postmortem investigations showed decreased GLUT1 and GLUT3 protein levels in AD brains (Simpson et al., 1994). Therefore, reductions in GLUT1 and GLUT3 levels might contribute to neuronal dysfunction and neurodegeneration (Winkler et al., 2015). Also, vascular endothelial growth factor (VEGF), has been proved tightly associated with glucose uptake. The ablation of VEGF reduces brain glucose uptake and cognitive function (Jais et al., 2016). However, increased VEGF expression can enhance GLUTs expression and cerebral glucose metabolism (Yeh et al., 2008; Jais et al., 2016; Wang et al., 2018).

Neurons process glucose directly through glycolysis. Hexokinase 1 (HK1) and Hexokinase 2 (HK2) are the ubiquitously expressed phosphofructokinase, which catalyzes the rate-limiting and first irreversible step of glycolysis (Zheng et al., 2016). HK1 and HK2 may be causally linked to neurodegenerative disorders, the activity of HK declines quickly in the elderly and AD patients (Harris et al., 2014; An et al., 2018). Besides, the transcriptional coactivator proliferator-activated receptor gamma coactivator 1-alpha (PGC1-α), which plays a key role in HK activity regulation, has also decreased in AD (Qin et al., 2009).

DNA repair deficiency contributes to mitochondrial dysfunction and deteriorates the glucose metabolism (Demarest et al., 2020). MutY homolog (MUTYH), MutT Homolog 1 (MTH1) and Oxoguanine DNA glycosylase 1 and 2 (OGG1/2) are crucial DNA repair enzymes, the oxidative DNA damage can be minimized by the functions of MUTYH with the mispaired bases, MTH1 with oxidized dNTPs and OGG1/2 with the 7,8-dihydro-8-oxoguanine (8oxoG). Increasing evidence indicates a decreased level of MTH1 and OGG1/2 in AD brains (Sliwinska et al., 2016; Abolhassani et al., 2017).

Recent studies have identified HIF-1α as an important transcription factor in glucose metabolism *via* regulating key factors involved in glucose uptake and glycolysis (Ferrara and Davis-Smyth, 1997; Harris et al., 2014). GLUT1 and GLUT3 are two important HIF-1α-regulated genes (Chen et al., 2001; Hayashi et al., 2004). Liu et al. (2008) revealed that decreased GLUT1 and GLUT3 may result from downregulated HIF-1α levels in AD brains, and it has strong connections with abnormal hyperphosphorylation of tau. HIF-1α also regulates GLUT4, and correlative research reported that HIF-1α reduction impaired GLUT4 translocation and glucose uptake (Sakagami et al., 2014). VEGF, which is the main factor involved in cerebral blood flow, plays a critical compensatory role to reinstate brain glucose metabolism (Jais et al., 2016). Interestingly, emerging evidence shows that the infusion of brain-derived neurotrophic factor (BDNF) improves glucose metabolism and cognitive function (Kuroda et al., 2003; Li et al., 2012; Kuipers et al., 2016). BDNF increased VEGF mRNA and protein expression *via* a HIF-1α signal transduction pathway (Lin et al., 2014), and the underlying mechanism was the activation of the BDNF/tyrosine Kinase receptor B (TrkB) pathway, which stimulate increases in HIF-1α (Nakamura et al., 2006; Helan et al., 2014; Jin et al., 2019).

Agents, such as iron chelators, effectively maintain HIF-1α levels in neurons (Guo et al., 2017). Therefore, we hypothesized that the maintenance of HIF-1α levels *via* the BDNF/TrkB pathway using iron chelators would be an effective way to attenuate glucose metabolism and abnormal tau pathology. α-Lipoic acid (LA) is a potent iron chelator and antioxidant that was found to be beneficial in the mitigation of oxidative stress, inflammation, ferroptosis, and tauopathy in our previous study (Zhang et al., 2018). Vassilopoulos and Papazafiri (2005) reported that LA maintained cardiomyocytes function and survival *via* stabilization of HIF-1α. However, no evidence of an association between LA and HIF-1α in AD has been reported. The present study first reported that LA supplementation effectively induced HIF-1α expression and alleviated cognitive deficits in Tau P301S mice (an AD mouse model) by reinstating glucose metabolism impairment *via* the BDNF/TrkB/HIF-1α signaling pathway.

## Materials and Methods

### Animals and Pharmacological Treatments

The P301S transgenic mice [B6C3-Tg (Prnp-MAPT*P301S) PS19 Vle/J] were originally obtained from the Jackson Laboratory (Bar Harbor, ME, USA), and housed in controlled temperature and natural light-cycle room. Twenty-one female mice were raised in filtered cages with free food and water until 5 months, then they were randomly assigned to three treatment groups (7 mice/group) corresponding to vehicle control, 3 mg/kg LA, and 10 mg/kg LA (T5625, Sigma–Adrich, St. Louis, MO, USA). LA was administered by intraperitoneal injection once per day, and the vehicle control mice received an identical volume of physiological saline. After continuously injected for 3 days, the animals got 1 day off, and treatment was stopped when cumulative injection add up to 2 months. The dosage and treatment protocol of LA was determined according to previous studies (Maritim et al., 2003; Zhao et al., 2015; Mahboob et al., 2016). During chronic treatment with LA, the body weight and health conditions were monitored daily. The wild type (WT) mice used as control were purchased from HFK bioscience company (Beijing, China) on a C57BL/6 background. All experiment procedures were accordance with the National Institutes of Health guidelines and approved by the Laboratory Animal Ethical Committee of China Medical University.

### Tissue Preparation

When all the treatment finished, mice (8-month-old) were euthanized by carbon dioxide euthanasia and perfused by physiological saline. The brain tissues were immediately taken from mice and cut into separate hemispheres on ice. The cerebral cortex was dissected from left hemisphere and stored in −80°C refrigerator for Western blot, RT-PCR, and hexokinase activity analysis. The right hemisphere was fixed in 4% paraformaldehyde at 4°C overnight and dehydrated in 30% sucrose until sinking to the bottom, then cut into frozen coronal sections (10 μm) for immunohistochemistry.

### Quantification of Hexokinase Activity

Hexokinase (HK) activity assay was performed using a hexokinase activity colorimetric kit (Solarbio, BC0740, China) according to the manufacturer’s protocol. In brief, the cortex of brain tissues was homogenized in extraction buffer on ice. Then samples were centrifuged at 8,000× *g* for 10 min at 4°C to obtain a supernatant. Forty microliter supernatant was loaded with the preheated reaction mix (37°C) and read the initial absorbance (340 nm) at 20 s with a microplate reader (BioTek, Cytation5, USA), then the mixture was incubated in 37°C water bath for 5 min and read the absorbance (340 nm) at 5 min 20 s. The activity of HK was calculated according to the formula in the manufacturer’s protocol.

### Western Blotting Analysis

Protein extracts from WT and P301S transgenic mouse cortex tissues, and the Western blots, were prepared as described previously (Zhang et al., 2018). In brief, samples were sonicated in a well-mixed buffer which adds phosphatase inhibitor cocktail, protease inhibitor cocktail, sodium fluoride, and phenylmethanesulfonyl fluoride to RIPA lysis buffer in the proportion of 1:100. After incubated 1 h on ice, all samples were centrifuged at 13,684× *g* for 25 min at 4°C and the supernatants were collected. The nuclear and cytosolic protein fractions were separated using a Nuclear Protein Extraction Kit according to the manufacturer’s instructions (Solarbio, R0050, China). The protein concentrations of all samples were determined by BCA kits (Beyotime, P0010S, China).

Proteins (30 μg) were separated using 10% or 12% SDS-PAGE as previously described (Zhang et al., 2018) and transferred onto polyvinylidene difluoride (PVDF) membranes (Millipore, IPVH00010, Darmstadt, Germany). Membranes were blocked with 5% skim milk and incubated overnight at 4°C with the follwing primary antibodies: rabbit anti-HIF-1α (1:2,000; Thermo Fisher Scientific, PA316521, USA), rabbit anti-BDNF (1:1,000; Santa Cruz Biotechnology, sc-546, USA), rabbit anti-p-TrkA/B (1:2,000; Thermo Fisher Scientific, MA5-14926, USA), rabbit anti-TrkB (1:2,000; Cell Signaling Technology, 4638T, USA), rabbit anti-GLUT1 (1:2,000, BIOSS, bs-20173R, China), mouse anti-GLUT3 (1:1,000; Santa Cruz Biotechnology, sc-74497, USA), rabbit anti-GLUT4 (1:2,000, BIOSS, bs-0384R, China), rabbit anti-hexokinase II (1:2,000; Cell Signaling Technology, C35C4, USA), rabbit anti-hexokinase II (1:2,000; Cell Signaling Technology, C64G5, USA), rabbit anti-HO-1 (1:2,000; Thermo Fisher Scientific, PA5-27338, USA), rabbit anti-VEGF (1:2,000; Cell Signaling Technology, 2463S, USA), rabbit anti-PGC-1α (1:1,000; Cell Signaling Technology, 2178S, USA), mouse anti-MUTYH (1:1,000; Santa Cruz Biotechnology, sc-374571, USA), mouse anti-OGG1/2 (1:1,000; Santa Cruz Biotechnology, sc-376935, USA), rabbit anti-MTH1 (1:1,000; Santa Cruz Biotechnology, sc-67291, USA), mouse anti-β-actin (1:10,000; Sigma–Aldrich, A1978, USA). After rinsed with TBST (leagene, pw0020, Beijing, China), the membranes were incubated with horseradish peroxidase-labeled secondary antibodies (1:10,000; Thermo Fisher Scientific, A27035 and A27022, USA) for 1 h at room temperature. Membranes were incubated with enhanced chemiluminescence (ECL) kits (Tanon, 180-5001B, China) for blot signal detection and visualized using Chem Doc XRS with Quantity One software (Tanon, 5500, China). Protein levels were normalized to actin, and phosphorylated proteins were normalized to their respective total protein amounts. Quantification and analyses were performed using ImageJ and Prism software.

### Immunohistochemistry and Confocal Microscopy

The sections were repaired in L.A.B solution (Polyscience, 24310-500, USA) and blocked with goat serum for 1 h at room temperature. After incubating with primary antibodies overnight at 4°C, the sections were rinsed with PBS, incubated with a secondary fluorescent-labeled antibody, and stained with DAPI (Solarbio, C0065, China). Then the sections were sealed in an anti-fade mounting medium. Immunohistochemical detection was performed using a fluorescence microscope (Leica, SP8, Germany). The following primary antibodies were used in the present study: rabbit-anti-BDNF (1:200; Sigma–Aldrich, SAB2108004, USA), mouse-anti-NeuN (1:200; Abcam, ab104224, UK), rabbit-anti-TrkB (1:50; Bioss, bs-0175R, China) and rabbit-anti-HIF-1α (1:100; Novus, NB100-479, Japan).

### Real-Time PCR

Total RNA of the cortex was extracted using Trizol (Thermo Scientific, 10296010, USA), and the RNA concentration was quantified using a Nanodrop at 260/280 nm (Thermo Scientific, ND1000, USA). One microgram total RNA was reverse transcribed to cDNA according to the manufacturer’s recommendations using GoTaqR 2-Step RT-qPCR System (Promega, A5001, USA). The generated cDNA was used for subsequent PCR reactions. All PCR reactions performed in a total volume of 20 μl: DNA polymerase activation for 10 min at 95°C, then 40 cycles of denaturing for 30 s at 95°C and annealing and extension for 30 s at 58°C. All applied primer pairs were listed in Supplementary Table S1. The mRNA expression levels were calculated using ^ΔΔ^Ct (threshold cycle, Ct) values normalized to GAPDH.

### Statistical Analysis

The difference between groups was analyzed by the unpaired two-tailed Student’s *t*-test for two data sets, or the one-way analysis of variance (ANOVA) for the three data sets by Graphpad Prism 5.0 (Graphpad Software, USA). Before applying the one-way ANOVA analysis, the Gaussian distribution of data was analyzed by the Kolmogorov–Smirnov method. All data are presented as mean ± SEM. Significance was set at *p* < 0.05.

## Results

### LA Treatment Improves Glucose Metabolism Deficiency in P301S Mice Brains

**Figure 1:**
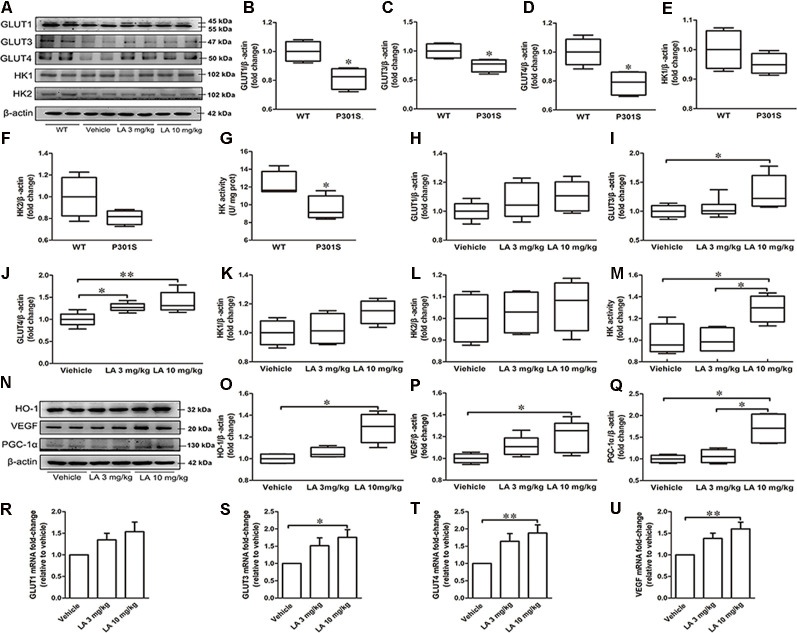
α-Lipoic acid (LA) improved glucose metabolism deficiency in P301S mice. **(A)** Representative western blots showed the expression levels of glucose transporter 1 (GLUT1), GLUT3, GLUT4, hexokinase-1 (HK-1), and HK2. **(B–F)** Quantified results of GLUT1, GLUT3, GLUT4, HK1, and HK2 levels between wild type (WT) and P301S mice. β-actin served as an internal loading control. **(G)** HK activity between WT and P301S mice. **(H–L)** Quantified results of the levels of GLUT1, GLUT3, GLUT4, HK-1, and HK2 among vehicle, LA 3 mg/kg, and 10 mg/kg groups. β-actin served as an internal loading control. **(M)** HK activity among vehicles, LA 3 mg/kg, and 10 mg/kg groups. **(N–Q)** Representative western blots and quantified results of the levels of heme oxygenase-1 (HO-1), vascular endothelial growth factor (VEGF), and proliferator-activated receptor gamma coactivator 1-alpha (PGC-1α). β-actin served as an internal loading control. **(R–U)** mRNA level of GLUT1, GLUT3, GLUT4, and VEGF. Values are represented as the means ± SEM (*n* = 7). *p < 0.05, **p < 0.01.

To see whether glucose metabolism was altered in P301S mice brains, we monitored glucose uptake and metabolism-related protein expression in P301S and WT mice. As a result, we found that GLUT1, GLUT3, GLUT4 expression level, and HK activity were decreased in P301S mice brains by ~19%, 25%, 22%, and 20%, respectively (Figures 1A–G). Whereas no significant alteration was observed in HK1 and HK2 protein level (Figures 1A–G). These data suggested glucose metabolism deficiency might exist in P301S mice.

To test if the dysregulated glucose metabolism can be restored by LA treatment, we first examined the glucose uptake related protein level. As shown in Figure 1A, the protein expression level of GLUT3 was significantly increased by ~32% in 10 mg/kg LA treated group when compared to the vehicle group (Figures 1A,I). The protein expression level of GLUT4 was significantly increased in both 3 mg/kg (~28% of vehicle control) and 10 mg/kg (~39% of vehicle control) LA treated groups (Figures 1A,J). In line with this, the mRNA level of GLUT3 (Figure 1S, ~72% of vehicle control) and GLUT4 (Figure 1T, ~37% of vehicle control) are also increased in 10 mg/kg LA treated group. However, there were no significant differences in GLUT1 protein or mRNA levels among the three groups (Figures 1A,H,R).

VEGF is an effective compensatory factor that facilitates glucose uptake and metabolism. To investigate whether LA treatment altered VEGF expression, Western blotting and RT-PCR were used. Our data showed a significant elevation in VEGF protein (Figures 1N,P, ~20% of vehicle control) and mRNA levels (Figure 1U, ~60% of vehicle control) in the 10 mg/kg LA-treated group. VEGF is regulated by HO-1. We further examined HO-1 expression using Western blotting, and the HO-1 expression level was significantly increased by ~28% in P301S mouse brains after 10 mg/kg LA treatment (Figures 1N,O).

Next, we evaluated the HK expression level and activity in the cortex of P301S mice after LA treatment. We found LA treatment did not change the HK1 and HK2 expression levels (Figures 1A,K,L). However, the HK activity was significantly increased in 10 mg/kg LA treated group (Figure 1M, ~29% of vehicle control). Additionally, we also examined the PGC-1α level, which involved in the regulation of HK activity. 10 mg/kg LA treatment dramatically upregulated the expression of PGC-1α (Figures 1N,Q, ~70% of vehicle control, ~64% of 3 mg/kg LA group). Collectively, the glucose metabolism deficiency in P301S mice was improved by LA treatment in a dose-dependent manner.

### LA Treatment Induces the Expression of DNA Repair Enzymes in P301S Tau Mice

**Figure 2:**
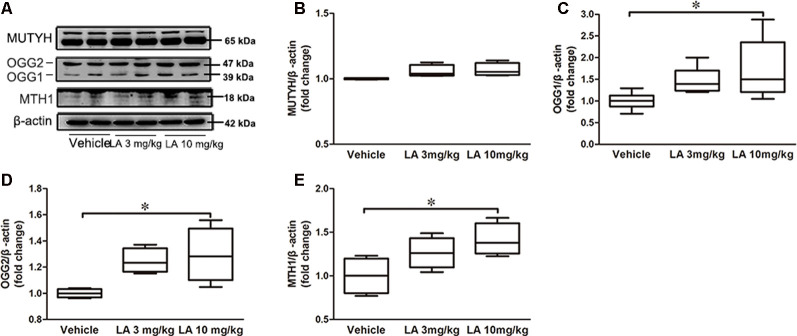
LA treatment promotes DNA repair by upregulating MutT H homolog (MTH1) and Oxoguanine DNA glycosylase 1 and 2 (OGG1/2). **(A)** Representative western blots showed the expression levels of MutY homolog (MUTYH), OGG1/2, MTH1. **(B–E)** Quantified results of the levels of MUTYH, OGG1/2, MTH1. β-Actin served as an internal loading control. Values are represented as the means ± SEM (*n* = 7). *p < 0.05.

DNA repair deficiency is tightly correlated with glucose metabolism. Therefore, we measured MTH1, OGG1/2, and MUTYH expression levels in the brain, which are the three main enzymes involved in the repairing of DNA oxidative damage (Nakabeppu, 2014). No significant differences in the MUTYH level were observed between LA-treated and vehicle groups (Figures 2A,B). However, OGG1, OGG2 and MTH1 levels were increased by ~47%, 73%, and 41%, respectively, in 10 mg/kg LA treated group (Figures 2A,C–E). The results indicated upregulated DNA repairment by 10 mg/kg LA administration thus contributing to the glucose metabolism restoration.

### LA Treatment Upregulates HIF-1α Expression *via* the BDNF/TrkB Pathway

**Figure 3:**
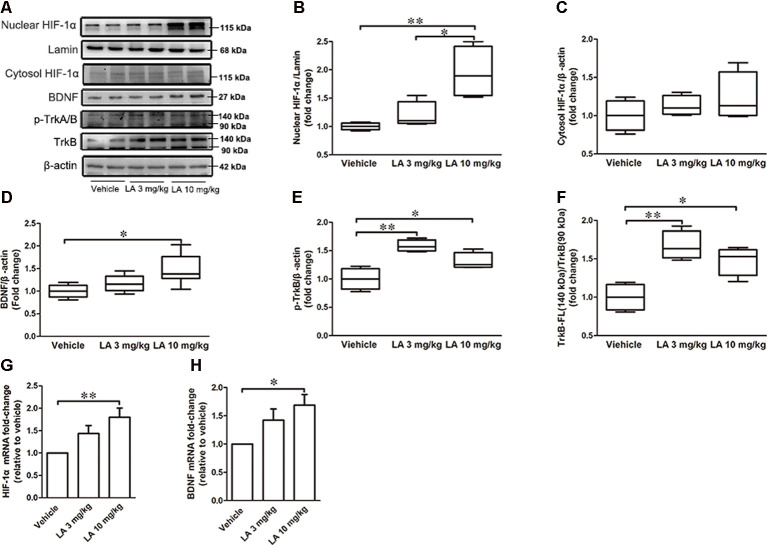
LA mediated the upregulation of hypoxia-inducible factor-1α (HIF-1α) expression *via* brain-derived neurotrophic factor/tyrosine Kinase receptor B (BDNF/TrkB) pathway. **(A)** Western blot results showed the protein expression levels of nuclear HIF-1α, Lamin, and cytosol HIF-1α, BDNF, p-TrkA/B, TrkB. **(B)** Quantified results of nuclear HIF-1α levels. Lamin served as an internal loading control. **(C–F)** Relative protein levels of cytosol HIF-1α, BDNF, p-TrkB, TrkB-FL which were established after normalization to β-actin. **(G,H)** mRNA levels of HIF-1α and BDNF. Values are represented as the means ± SEM (*n* = 7). *p < 0.05, **p < 0.01 compared with the vehicle group.

**Figure 4:**
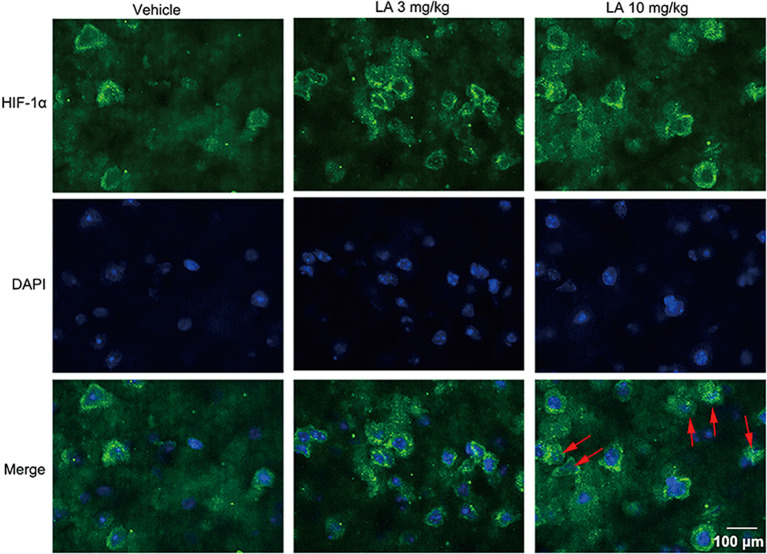
LA induces HIF-1α expression and nuclear translocation in cortex neurons. HIF-1α activity and its cellular localization in the cortex was determined by immunofluorescence staining. HIF-1α was predominantly found in the cytosol and nucleus of cortex neurons in 10 mg/kg LA-treated P301S mice. The red arrowheads point out the translocation of HIF-1α into the nucleus. Scale bar equals 100 μm.

To assess the effects of LA treatment on HIF-1α, we used Western blotting, immunohistochemistry, and RT-PCR to evaluate HIF-1α expression levels. 10 mg/kg LA treatment promoted HIF-1α translocation from the cytosol into nuclear and dramatically upregulated the nuclear HIF-1α expression levels (Figures 3A,B, ~95% of vehicle control, ~75% of 3 mg/kg LA group). Also, we observed an increasing trend of cytosol HIF-1α after LA treatment but without significant change (Figures 3A,C). The mRNA levels of HIF-1α were also dramatically upregulated by 10 mg/kg LA treatment (Figure 3G, ~94% of vehicle control). Likewise, we also observed that 10 mg/kg LA treatment significantly increased HIF-1α expression and nucleus translocation in the cortex of P301S mice by immunofluorescence staining (Figure 4).

**Figure 5:**
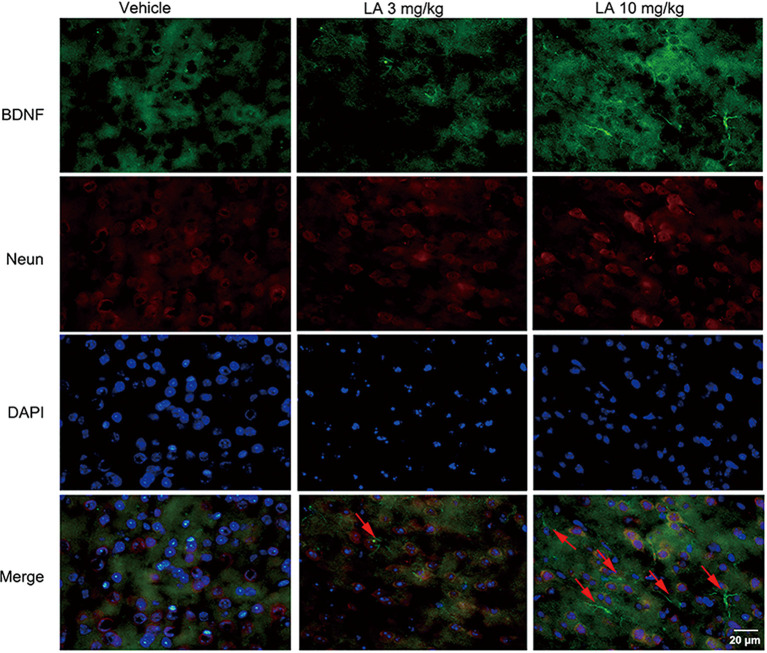
Effect of LA on BDNF expression in the P301S mice cortex. Double immunofluorescence of frozen brain slices using BDNF (green) and Neun (red). A stronger BDNF immunoreactivity was observed in both 3 mg/kg and 10 mg/kg LA-treated group. The red arrowheads point out the astrocytes-derived BDNF. Scale bar represents 20 μm.

To gain insight into the mechanisms underlying the elevated HIF-1α expression level, the BDNF/TrkB pathway was analyzed. 10 mg/kg LA administration significantly enhanced the protein and mRNA levels of BDNF by ~48% and 69%, respectively, in P301S mice brains (Figures 3A,D,H). BDNF levels were also measured using immunofluorescence staining in the cortex of P301S mice, and the results showed a dramatic increase in BDNF expression of 10 mg/kg LA-treated group (Figure 5). Notably, we also observed astrocyte-derived BDNF in 10 mg/kg LA-treated group (Figure 5, indicated by the red arrows), but these changes were not observed in the vehicle group (Figure 5). These results suggested that LA upregulates neuron- and astrocyte-derived BDNF expression levels in a dose-dependent manner.

**Figure 6:**
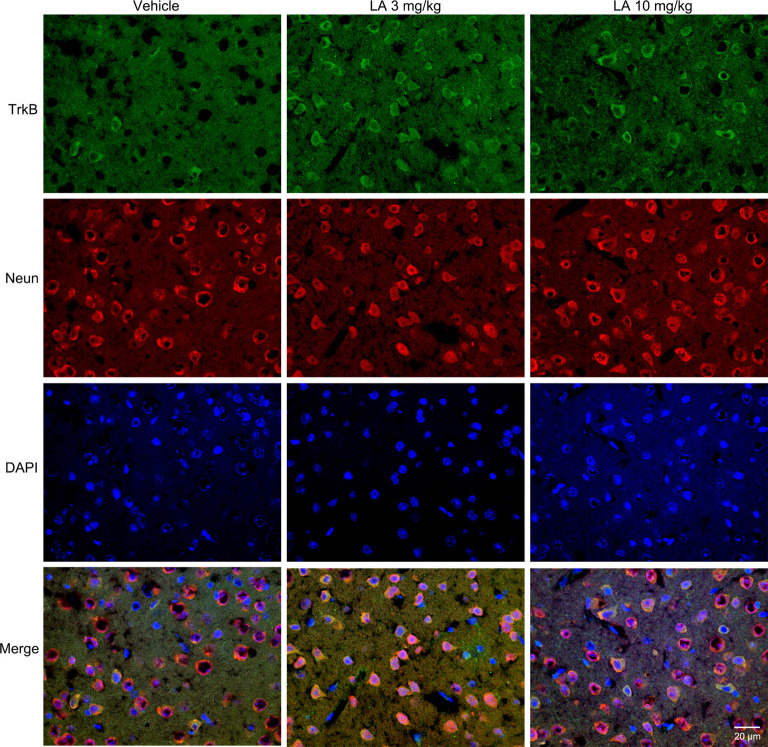
Effect of LA on TrkB expression in the P301S mice cortex. Double immunofluorescence of frozen brain slices using TrkB (green) and Neun (red). A stronger TrkB immunoreactivity was observed in both 3 mg/kg and 10 mg/kg LA-treated group. Scale bar represents 20 μm.

TrkB is the main receptor for BDNF, which has two main isoforms, the full-length form (TrkB-FL, 140 kDa) and the truncated form (tTrkB, 90 kDa). Binding of BDNF to TrkB-FL triggers BDNF dimerization and TrkB activation (autophosphorylation). Therefore, we analyzed the expression levels of TrkB-FL and tTrkB using Western blotting. Significantly increased levels of TrkB-FL were observed in the 3 mg/kg (~67% of vehicle control) and 10 mg/kg (~48% of vehicle control) LA-treated group (Figures 3A,F). We also examined phosphorylated TrkB (p-TrkB, Tyr516) expression levels in P301S mouse brains. The significant upregulation of p-TrkB (Tyr516) level was observed in 3 mg/kg (~58% of vehicle control) and 10 mg/kg (~31% of vehicle control) LA-treated mice (Figures 3A,E). Besides, TrkB levels were also determined using immunofluorescence staining in the cortex of P301S mice, and the results showed a significant increase in TrkB expression of both 3 mg/kg and 10 mg/kg LA-treated group (Figure 6), which was consistent with the results of WB.

## Discussion

It is widely known that LA has a variety of properties that can interfere with the pathogenesis or progression of AD. The efficacy of LA on AD has been approved by several clinical trials (Hager et al., 2001, 2007; Salinthone et al., 2010; Fava et al., 2013; Shinto et al., 2014). Now, a growing body of evidence suggests that glucose hypometabolism may be a key player in AD (Kuehn, 2020). The anti-AD properties of LA include stimulating glucose uptake and utilization (Maczurek et al., 2008), however, the underlying mechanism remains unclear. In the present study, we showed that LA treatment could restore the glucose uptake and glycolysis in P301S mice by upregulating BDNF/TrkB/HIF-1α pathway.

Glucose uptake and utilization will significantly influence the survival and normal function of neurons. GLUT1, GLUT3, and GLUT4 are highly expressed in the brain (Marko et al., 2020) and correlates positively with cerebral glucose utilization due to a high affinity for glucose (Jin et al., 2013). Researches showed the lower levels of these GLUTs are associated with more severe AD pathology (An et al., 2018), and the ablation of GLUT3 and GLUT4 in mouse brain triggered glucose uptake deficits and synaptic deficits (Ashrafi et al., 2017; Reno et al., 2017). Glycolysis is the first step of glucose metabolism and provides metabolic precursors for neuronal energy production. HK1 and HK2 are the main rate-limiting enzymes in glycolysis and the decreased HK activity has been reported in AD brains (Harris et al., 2014). In the present study, we assessed the glucose metabolism in the P301S transgenic mouse, which expressed 5-fold higher human tau protein than the endogenous mouse tau protein in the brain. We found altered glucose metabolism in P301S mice brains accompanied downregulated glucose transporters (GLUT1, GLUT3, and GLUT4) and HK enzyme activity, which indicated the glucose metabolism deficiency in P301S mice.

Interestingly, several studies suggest LA could improve glucose metabolism disorder in several metabolic syndromes (Agathos et al., 2018; Aslfalah et al., 2019; Gosselin et al., 2019). However, researches on the effect of LA in AD glucose metabolism is still rarely reported at present. In the present study, we found 10 mg/kg LA could significantly increase both protein and mRNA level of the neuronal glucose transporter (GLUT3 and GLUT4) in the cortex of P301S mice, which suggested that LA reinstated glucose uptake in neurons of P301S mice, which were consistent with previous studies (Bitar et al., 2004; Maczurek et al., 2008). Besides directly upregulating GLUTs, our data also suggest that LA treatment indirectly contributes to glucose uptake *via* VEGF induction in a dose-dependent manner. VEGF is required to maintain brain glucose uptake across the blood-brain barrier (Schüler et al., 2018). We further analyzed the mechanisms underlying the upregulated effect of LA on VEGF by examining the expression of the main regulator, HO-1, in P301S mouse brains. We found that LA treatment effectively induced HO-1 expression, which suggested that LA compensated glucose uptake deficiency *via* VEGF by upregulating HO-1.

Glucose uptake is upstream of fuel supplement for neurons, and the subsequent glycolysis is the major pathway for neuronal energy production. Researches showed glycolysis upregulation is neuroprotective as a compensatory mechanism in neurodegenerative disorders (Manzo et al., 2019). Our results indicated that the activity of HK was significantly increased after 10 mg/kg LA treatment. Consistent with the results, the level of PGC-1α was upregulated in LA treated mice, which involved in the regulation of HK activity. This is following another study that shows LA improves glycolysis deficiency might include PGC-1α-mediated increases in HK expression and activity (Fu et al., 2014; Yang et al., 2014).

DNA repair deficiency is believed to deteriorate the glucose metabolism by driving mitochondrial dysfunction (Demarest et al., 2020). Herein, we evaluated the expression levels of three important DNA repair enzymes, MTH1, OGG1/2, and MUTYH (Nakabeppu, 2014). We observed increased OGG1/2 and MTH1 expression in the LA-treated group, but there were no significant changes in the MUTYH level. MTH1 and OGG1/2 can suppress neurodegeneration by preventing 8-oxoG accumulation in cellular DNA, and mutant mice lacking MTH1 and OGG1 exhibit severe neurodegeneration (Sheng et al., 2012). The OGG1 splice variant (39 kDa) is the most prevalent form, and it localizes to the nucleus. The OGG2 splice variant (47 kDa) is targeted to the mitochondria. Our results indicated an upregulation of DNA repair in the nucleus and mitochondria *via* the induction of OGG1/2 and MTH1 expression, which suggested the restoration effect of LA on glucose metabolism.

LA treatment increased glucose availability in the cortex of P301S mice, which may be involved in the regulation of HIF-1α. HIF-1α is an important transcription factor that regulates glucose uptake and glycolysis at the transcriptional level (Huang et al., 2012; Harris et al., 2014; Masoud and Li, 2015). Several studies have shown that HIF-1α upregulates glucose transporters (GLUT1, GLUT3, GLUT4; Toberer et al., 2020), glycolysis enzyme (HK2; Cao et al., 2020; Shen et al., 2020), pro-angiogenic factors (HO-1, VEGF; Choi et al., 2006). Whereas, downregulation of HIF-1α suppressed the target genes and protein expressions, such as GLUT1, GLUT3, and HK2 (Liu et al., 2008; Lee et al., 2019). In the present study, LA treatment effectively triggered the upregulation and nuclear translocation of HIF-1α, which activated the transcription of several downstream target genes (Morand et al., 2019). As expected, we observed an enhanced level of GLUT3, GLUT4, VEGF, HO-1, HK activity in 10 mg/kg LA treatment group.

Several pieces of research indicate that activation of the BDNF/TrkB pathway upregulates HIF-1α expression even under normoxic conditions (Nakamura et al., 2006; Helan et al., 2014; Lin et al., 2014; Jin et al., 2019). BDNF is essential for the maintenance of cortical neurons, whose downregulation contributes to the initial loss of short-term memory in AD (Giuffrida et al., 2018). Our data showed that 10 mg/kg LA administration significantly elevated BDNF expression levels both in neurons and astrocytes. The neurotrophic function of BDNF is mainly regulated by the TrkB. TrkB has two main isoforms: the full-length form (TrkB-FL) and the truncated form (tTrkB). The binding of BDNF to TrkB-FL is necessary for the receptor homodimerization, which initiates tyrosine residue phosphorylation and translocation of the BDNF/p-TrkB complex and regulation of the downstream signaling pathways. The results showed a significant increase of TrkB-FL expression level after 10 mg/kg LA administration. Ca^2+^-dependent calpain is the major mechanism of TrkB-FL cleavage (Tejeda et al., 2019). Our previous study found that LA treatment significantly decreased Ca^2+^ content and calpain expression levels (Zhang et al., 2018), which is consistent with the decreased TrkB-FL cleavage in the present study. The phosphorylation of tyrosine sites of TrkB contributes to BDNF signal transduction (Huang and Reichardt, 2003), and we observed significantly increased levels of p-TrkB (Tyr 516) in 10 mg/kg LA-treated group. Collectively, our results suggest that chronic LA administration might enhance HIF-1α expression *via* the BDNF-TrkB signaling pathway.

## Conclusion

**Figure 7:**
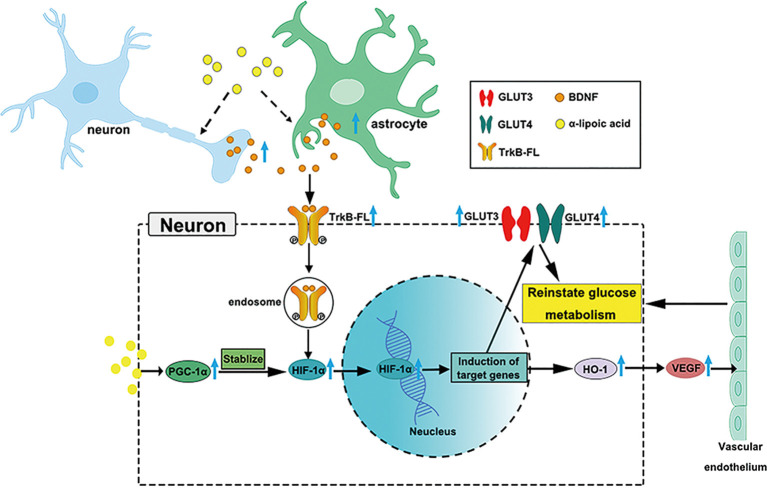
Schematic model of the possible mechanisms of BDNF/TrkB/HIF-1α involvement in maintaining brain glucose metabolism in P301S mice. Chronic LA treatment induces increased BDNF expression level in neurons and astrocytes in P301S mice brain, then BDNF binding to TrkB-FL induces the activation of TrkB-FL (autophosphorylation of tyrosine sites), BDNF and p-TrkB-FL complex translocate from the cellular membrane through forming endosomes. The complex upregulates HIF-1α protein level and contributes to HIF-1α nucleus translocation, inducing the downstream target genes expression, such as GLUT3, GLUT4, and HO-1, VEGF. The increased glucose transporters and vascular endothelium reinstate glucose metabolism in P301S mice. Also, LA administration upregulates PGC-1α expression and promotes the stabilization of HIF-1α protein level, couple with HIF-1α to induce the downstream target genes.

LA is used clinically to treat AD (Hager et al., 2007, 2010; Fava et al., 2013), but the growing knowledge we provide on glucose metabolism restoration by LA *via* the BDNF/TrkB/HIF-1α pathway in AD may support a novel therapy target for neurodegenerative diseases (Nakamura et al., 2006; Li et al., 2012; Lin et al., 2014; Jiao et al., 2016). Our study supports LA as a critical supplement to reinstate brain glucose metabolism, which is involved in the upregulated expression of GLUT3, GLUT4, VEGF, OGG1/2, MTH1, and HK activity. A schematic diagram of the role of LA in maintaining brain glucose metabolism is shown in Figure 7. The present study establishes LA as a therapeutic option in AD in the future.

## Data Availability Statement

All datasets generated for this study are included in the article/Supplementary Material.

## Ethics Statement

The animal study was reviewed and approved in accordance with the National Institutes of Health guidelines, and the experimental procedures were approved by Laboratory Animal Ethical Committee of China Medical University.

## Author Contributions

QA and CG conceived and designed the experiments. Y-HZ performed most of the experiments and wrote the manuscript. X-ZY, S-FX, Z-QP, L-BL, YY, Y-GF, ZW and XY contributed to generating and validating the mouse model. All authors contributed to the article and approved the submitted version.

## Conflict of Interest

The authors declare that the research was conducted in the absence of any commercial or financial relationships that could be construed as a potential conflict of interest.
