# Supplementary material for: α-Lipoic Acid Maintains Brain Glucose Metabolism via BDNF/TrkB/HIF-1α Signaling Pathway in P301S Mice
Source: Front Aging Neurosci. 2020 Aug 21;12:262. doi: 10.3389/fnagi.2020.00262 (PMC7471806; doi:10.3389/fnagi.2020.00262)
Supplement: Supplementary file 1 [file Data_Sheet_1.PDF]

Supplementary Material

1 Supplementary Figures and Tables

1.1 Supplementary Table

Table **1** Primer sequences

name	forward	reverse	size	species
Bdnf	5’- GTCACAGCGGCAGATAAA -3’	5’- ATTGGGTAGTTCGGCATT-3’	196	mouse
Hif-1α	5’- CAAGCCCTCCAAGTATGA -3’	5’-CCTTAGCAGTGGTCGTTT-3’	198	mouse
Vegf	5’- CTACTGCCGTCCGATTGAG-3’	5’-CTGGCTTTGGTGAGGTTTG -3’	196	mouse
Glut1	5’- CCCAGCCCTGCTACAGT-3’	5’- GGTCTCGGGTCACATCG-3’	137	mouse
Glut3	5’- CTCCTTGGCTTAACCATC-3’	5’-CTCCTGTGACATCCGAAC-3’	198	mouse
Glut4	5’- TGCTGCCCTTCTGTCCTG-3’	5’-CAACTTCCGTTTCTCATCCT-3’	146	mouse
Gapdh	5’- TGTTTCCTCGTCCCGTAG-3’	5’-CAATCTCCACTTTGCCACT-3’	108	mouse

The primer sequences used in RT-PCR.

1.2 Supplementary Figures

Fig.1

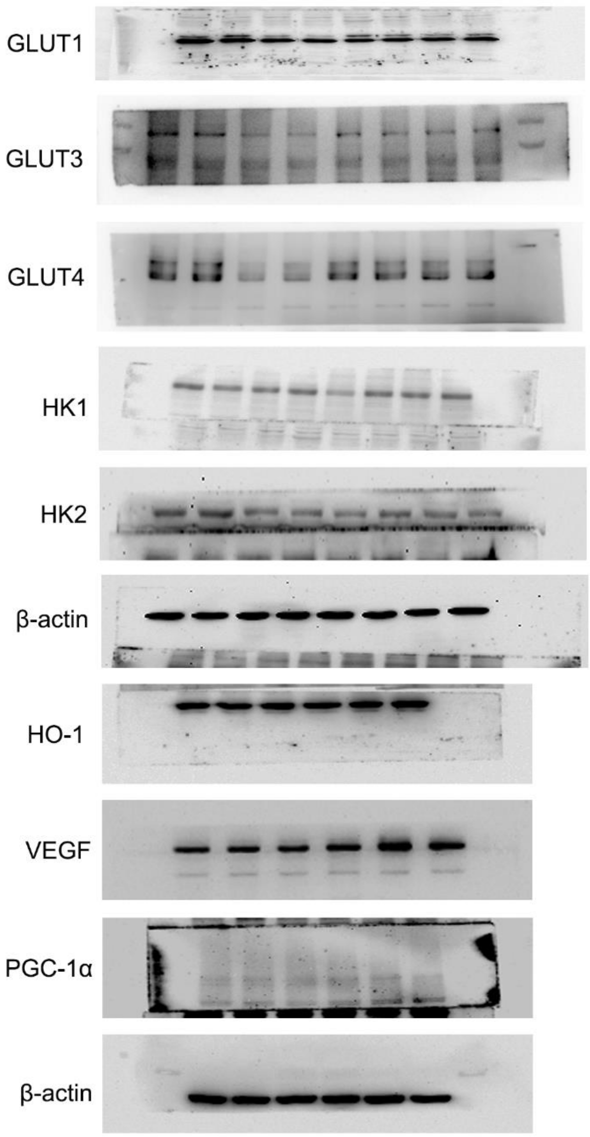

Supplementary Figure 1. The original image files for the blots in Figure 1.

Fig.2

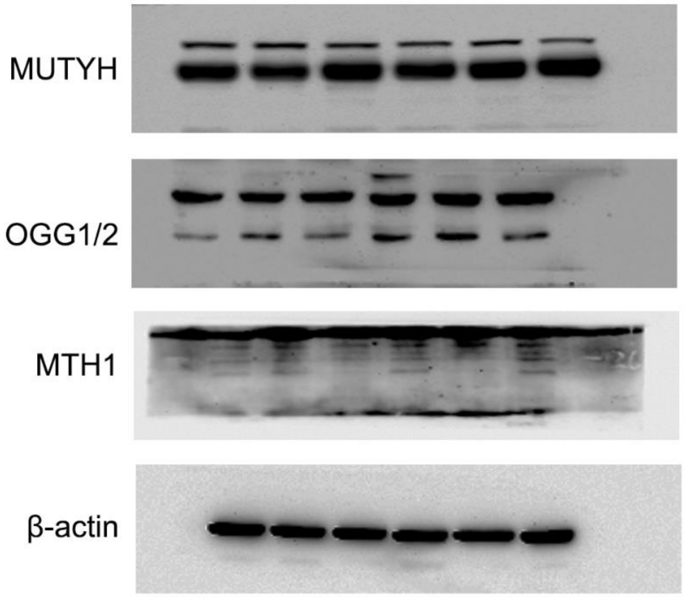

Supplementary Figure 2. The original image files for the blots in Figure 2.

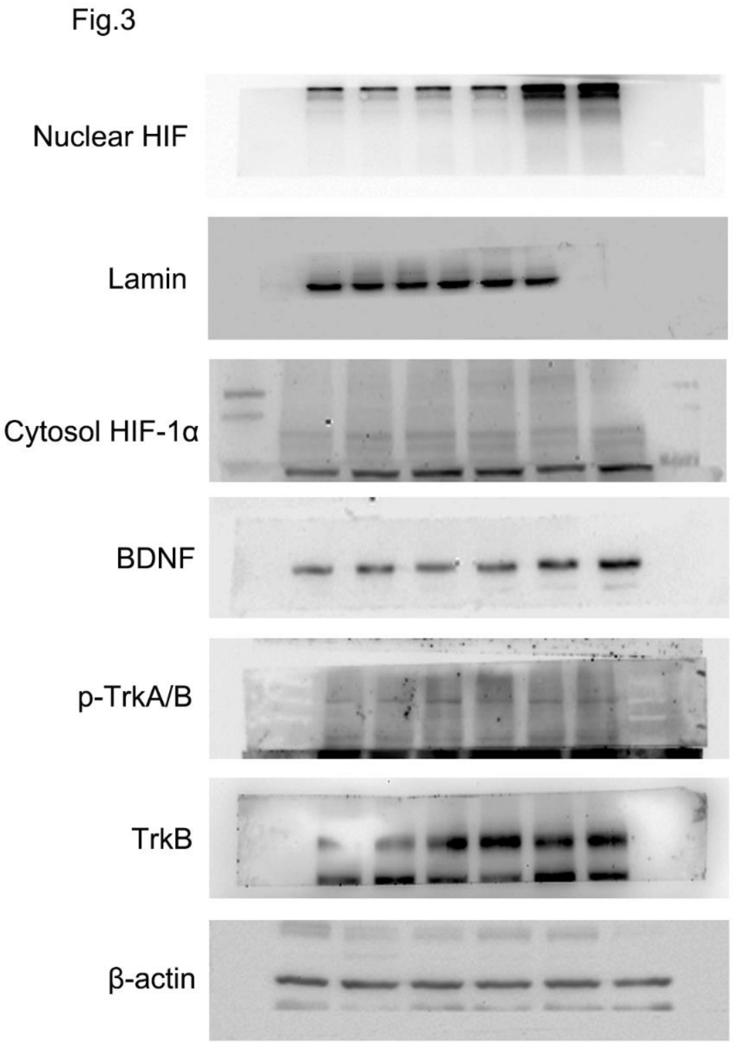

Supplementary Figure 3. The original image files for the blots in Figure 3.
